# A TaqMan-MGB real-time PCR for discriminating between MS-H-live vaccine and field *Mycoplasma synoviae* strains

**DOI:** 10.1128/spectrum.03591-23

**Published:** 2025-04-02

**Authors:** Ziqing Liu, Shouchang Zhou, Chenchen Meng, Doudou Ren, Weizhen Xiong, Guanhui Liu, Jinpeng Xu

**Affiliations:** 1 School of Life Science and Food Engineering, Hebei University of Engineering 117798, Hebei, China; 2 Huayu Agricultural Science and Technology Co., Ltd., Hebei, China; 3 Hebei Laying Hen Industrial Technology Research Institute, Handan, China; University of São Paulo, São Paulo, Brazil

**Keywords:** *Mycoplasma synoviae*, MS-H vaccine, real-time PCR, DIVA, *hlyC *gene

## Abstract

*Mycoplasma synoviae* (MS) is an important pathogen in the poultry industry and has caused significant economic losses. Worldwide, the use of live attenuated vaccine for the MS-H strain has increased to prevent MS infection. However, there is no test available to discriminate the MS-H vaccine strains from the MS strains that are causing field infection. In this study, a TaqMan-MGB real-time PCR method (qPCR) was established, validated, and evaluated to discriminate between MS-H-live vaccine and field strains based on nucleotide differences in the *hlyC* gene. The validation was performed for sensitivity and reproducibility by constructing recombinant plasmids. The limits of detection were 1.07 × 10^1^ copies/µL for the MS-H and 1.95 × 10^1^ copies/µL for field strains, respectively. The intra- and inter-assay results were less than 2.5% based on the reproducibility test. No cross-amplification signals from other common chicken pathogens were detected. Thus, our data indicated that this qPCR is sensitive, specific, and reproducible. In addition, 709 chicken clinical samples were used to evaluate this qPCR test. The results showed that positive signals could be detected from the chicken choanal cleft swabs and are 100% in concordance with the PCR sequencing method. To the best of our knowledge, we found for the first time that both L- and C-type field MS were present in flocks immunized against the MS-H vaccine strain during the validation process. In addition, this is the first report of a field strain of C-type in China.

**IMPORTANCE:**

*Mycoplasma synoviae* (MS) is an important pathogen in the poultry industry and has caused significant economic losses. Worldwide, an increasing number of farms are using the live attenuated vaccine MS-H strain to prevent MS infections. In order to monitor vaccinated and naturally infected flocks and to continue the MS control and eradication program, a differentiation of infected from vaccinated animals (DIVA) test for MS is urgently needed. We developed a TaqMan-MGB real-time qPCR (qPCR) method with a pair of primers and two competitive TaqMan-MGB probes. We performed an evaluation that can discriminate between the MS-H-live vaccine and field MS strains based on nucleotide differences in the *hlyC* gene. It has great sensitivity and reproducibility, and greater specificity than other methods which were established by SNP sites of the *obg* gene and *oppF* gene.

## INTRODUCTION

*Mycoplasma synoviae* (MS) primarily infects chickens and turkeys, causing subclinical respiratory diseases, infectious synovitis, eggshell apex abnormalities (EAA), and airsacculitis, resulting in significant economic losses to the poultry industry (1, 2). In recent years, MS has been reported in Australia, South America, Asia, Europe, and Africa, and its threat has exceeded that of *Mycoplasma gallisepticum* (MG), which has been classified as a notifiable pathogen by the World Organization for Animal Health (WOAH) (3–5).

Eradication strategies and vaccination are long-term solutions to control MS transmission (6). While eradication is the best way to prevent disease, it is very expensive and may be out of reach for farmers in many countries and regions that use serologic and molecular diagnostics, antibiotic control, and vaccination (7). Although countries such as the US and the UK still rely on eradication, vaccination is the method of choice for preventing and controlling MS infection in most countries. The current commercial live vaccines for MS are the temperature-sensitive (ts+) MS-H strain and the NAD-independent MS1 strain, of which the low-virulence MS-H-live vaccine has been used in commercial poultry flocks in different countries (8). Vaccination with the MS-H strain can help to prevent or alleviate clinical symptoms and reduce economic losses but cannot completely prevent infection with the field MS (9). Therefore, to monitor vaccinated flocks for freedom from infection and to continue our MS control and eradication program, a DIVA test for MS is urgently needed.

In this study, we describe the development, validation, and evaluation of a qPCR to discriminate between the MS-H-live vaccine strain and field MS based on the *hlyC* gene. The 5´-end conserved region of the variable lipoprotein hemagglutinin A (*vlhA*) gene from the clinical samples was cloned and sequenced since this is a common genomic target identified to date for MS genotyping. All samples were genotyped by proline-rich repeat (PRR) region length of *vlhA* gene analysis to validate the accuracy of qPCR results.

## RESULTS

### qPCR analytical sensitivity, amplification efficiency, and standard curve

**Fig 1:**
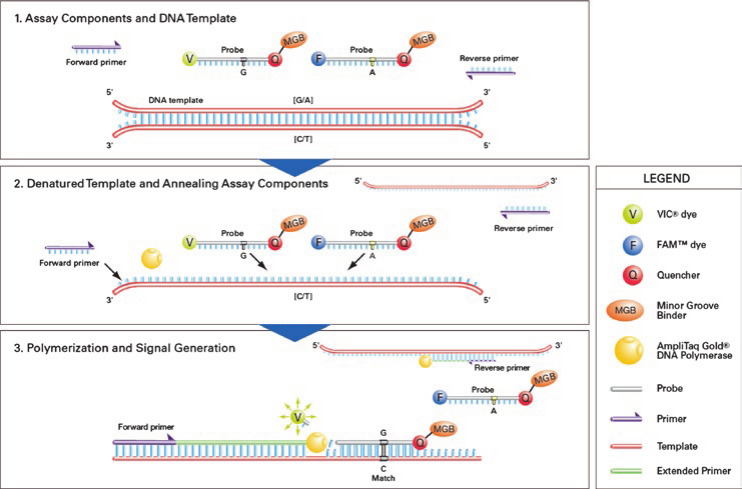
Working principle diagram of this qPCR.

**Fig 2:**
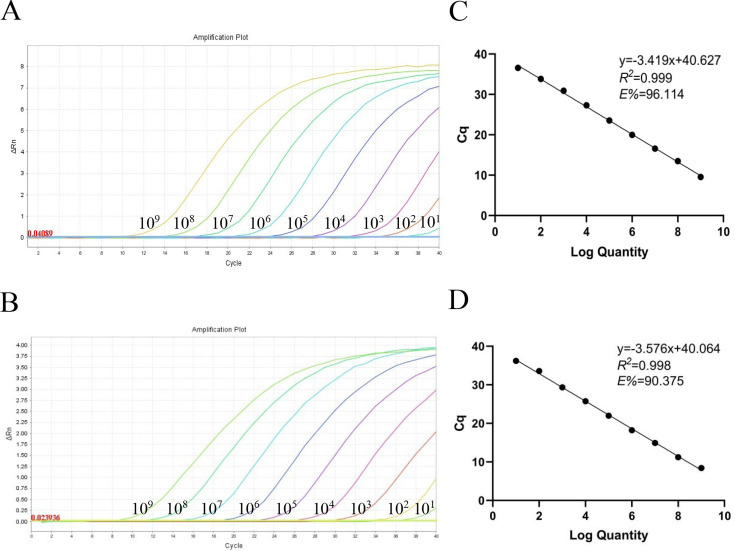
Preparation of the plasmid standards. (**A and B**) Amplification curves (X-axis: cycle, Y-axis: △Rn, fluorescence intensity) of MS-H and field MS for each standard plasmid with concentrations from 1 × 10^9^ copies/µL to 1 × 10^1^ copies/µL. (**C and D**) Standard curves of the standard plasmids of pMD19-T/MS-H and pMD19-T/XL3.

In this study, we developed qPCR to distinguish between the MS-H vaccine strain and field strains based on the SNP at 428 nt of the *hlyC* gene. The working principle diagram of this method is shown in Fig. 1 (10). The pMD19-T/MS-H and pMD19-T/XL3 were applied to evaluate the sensitivity and amplification efficiency of the assay. A standard curve was constructed, using the Cq value on the y-axis and the logarithm of the template concentration on the x-axis, and the Cq value showed a good linear relationship with the fluorescence signal as shown in Fig. 2.

The concentrations of the recombinant plasmid pMD19-T/MS-H and pMD19-T/XL3 were measured by microspectrophotometer as 33.44 ng/µL and 61.24 ng/µL, and the copy concentrations were calculated as 1.07 × 10^10^ copies/µL, 1.95 × 10^10^ copies/µL, and 10-fold dilution to 10° copies/μL. This qPCR was performed on the pMD19-T/MS-H and pMD19-T/XL3 at concentrations of 10^9^–10° copies/μL, and the LODs were 1.07 × 10^1^ copies/µL and 1.95 × 10^1^ copies/µL. Then, we constructed standard curves with the linear equations for the pMD19-T/MS-H and pMD19-T/XL3 combination were y = −3.419x + 40.627 (*R*^2^ = 0.999, *E* = 96.114%) and y = −3.576x + 40.064 (*R*^2^ = 0.998, *E* = 90.375%), respectively (Fig. 2). We tested 10^1^ copies/μL of positive plasmids 10 times each, and the Cq values were all in the range of 36–37, while 10° copies/μL were negative. Thereafter, we set the Cq value at 37 as the positive cut-off.

### Specificity and reproducibility assessment

**Fig 3:**
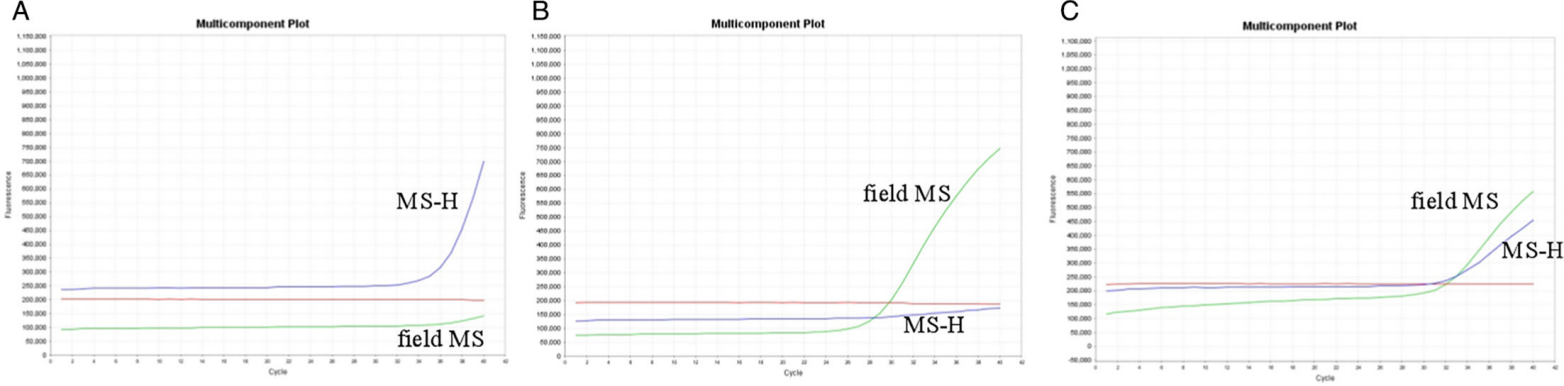
The result of both pMD19-T/MS-H and pMD19-T/XL3 is positive by qPCR. Blue line: FAM; red line: ROX; and green line: VIC. (**A**) The result of pMD19-T/MS-H is positive. (**B**) The result of pMD19-T/XL3 is positive. (**C**) The result of pMD19-T/MS-H and pMD19-T/XL3 is positive.

To evaluate the specificity of this qPCR, DNA or cDNA from 10 field MS and 7 other poultry pathogens, including MG, SE, AIV, NDV, IBV, *E. coli*, and Apg, were used as templates. These are common pathogens among poultry, which showed no signals by qPCR. The 10 field MS showed the VIC signal by qPCR. In addition, the qPCR showed the specificity of each probe and the opposite plasmid to show there is no cross-detection (Fig. 3).

The results of the reproducibility test showed that the coefficients of variation of the intra-assay and inter-assay Cq values of the qPCR ranged from 0.18% to 2.27%, which were less than 2.5% (Table 1), indicating that the established qPCR method has high reproducibility and reliability.

**TABLE 1:** Intra-assay and inter-assay comparisons for the qPCR

Plasmid	Concentration (copies/μL)	Intra-assay						Inter-assay					
		Cq value			Mean	SD	CV%	Cq value			Mean	SD	CV%
		1	2	3				1	2	3			
pMD19-T/MS-H	1.07 × 10^5^	23.55	23.57	23.27	23.46	0.17	0.71	23.53	23.01	23.12	23.22	0.27	1.18
	1.07 × 10^4^	27.31	26.44	27.03	26.93	0.44	1.64	26.74	26.83	26.76	26.78	0.05	0.18
	1.07 × 10^3^	30.90	29.79	30.57	30.42	0.57	1.87	29.80	30.10	29.81	29.90	0.17	0.57
pMD19-T/XL3	1.95 × 10^5^	21.98	22.27	21.99	22.08	0.16	0.75	23.07	22.42	22.06	22.52	0.51	2.27
	1.95 × 10^4^	25.76	25.89	25.84	25.83	0.07	0.25	26.39	26.41	25.47	26.09	0.54	2.06
	1.95 × 10^3^	29.36	29.85	29.55	29.59	0.25	0.84	29.46	29.51	30.42	29.80	0.54	1.81

### Analysis of clinical samples

A total of 709 choanal cleft swabs from 6 large-scale farms immunized against the MS-H strain were tested by qPCR. The choanal cleft swabs were vortexed in 400 µL of ddH_2_O, the supernatant was transferred to a new centrifuge tube, centrifuged at high speed (12,000 rpm, 4 min) to precipitate any MS present, 100 µL of the supernatant was retained, and DNA was extracted by the boiling method (100°C for 15 min) and stored at −20°C. The result of qPCR found that the positive rate of the MS-H strain was 65.87% (467/709), and the positive rate of field strains was 33.29% (236/709). The number of samples tested positive by qPCR for a single MS-H strain, single field strains, and mixed positives is shown in Table 2. Of the 709 clinical samples, the number of single-MS-H positive samples was 411, of which 335 were successfully sequenced; the number of single-field MS positive samples was 180, of which 146 were successfully sequenced; and the number of double-positive samples was 56, of which 56 were not sequenced. The PCR sequencing results were consistent with the qPCR assay results.

**TABLE 2:** Clinical sample testing

Farm	Number	qPCR			*vlhA* sequencing	
		Single-MS-H	Single-field MS	Mixed trace	MS-H	Field MS
A	100	1	61	23	1	47
B	99	54	26	8	42	19
C	120	0	93	25	0	80
D	130	120	0	0	108	0
E	140	126	0	0	96	0
F	120	110	0	0	88	0
Total	709	411	180	56	335	146

### Discovery of type C of MS field strains

**Fig 4:**
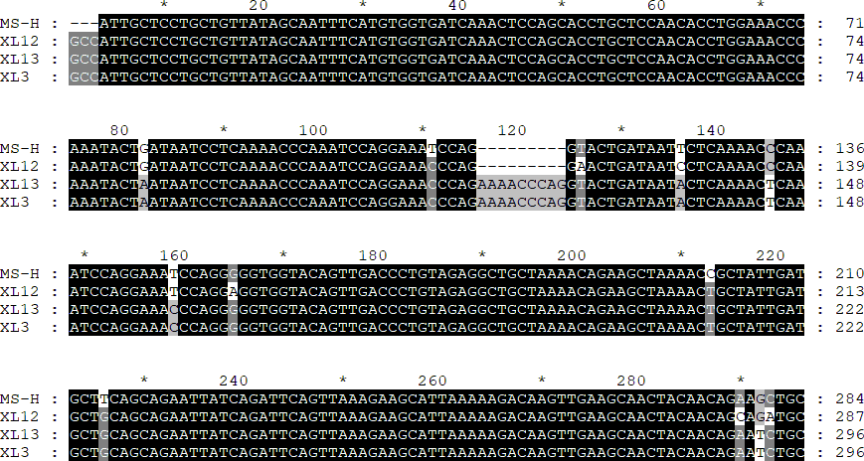
Sequence comparison of the MS-H strain (GenBank accession no. KX168666), XL12 (GenBank accession no. OR371720), XL13 (GenBank accession no. OR371721), and XL3 (GenBank accession no. OP850572). XL12, C-type field MS isolate. XL13, L-type field MS isolate.

Through PCR sequencing of clinical samples, we found that two MS field strains may exist in farm A, the C-type and L-type field strains. Although the MS-H strain was also C-type, the C-type field strain was significantly different in sequence from the MS-H strain. We uploaded the sequences of the C-type field strain (XL12) and the L-type field strain (XL13) to GenBank and obtained the accession numbers and compared the sequences of the MS-H strain, XL12, XL13, and XL3 using MEGA v7.0 and GeneDoc software (Fig. 4) By the blast analysis, the sequence of XL12 showed 99.44% similarity to the IZSVE/2958/MAV16/11 strain and the K1 strain at 100% coverage; the sequence of XL13 showed 100% similarity to the HN01 strain (GenBank accession no. CP034544) at 100% coverage.

## DISCUSSION

MS can be transmitted horizontally and vertically, and once infected, a bird is a lifelong carrier of the pathogen, causing enormous economic losses to the global poultry industry. The MS-H strain is a temperature-sensitive (ts+) vaccine produced by a chemical mutation of the Australian field strain 86079/7NS. The MS-H strain has been widely used in many countries to prevent infection with strong strains of MS (8, 11–14). MS-H colonizes in the upper respiratory tract of birds, which can induce a strong immune response after vaccination and effectively reduce the EAA caused by infection with field strains (8, 11–14). In a previous study, more than 9,773 broiler flocks in 16 provinces of China were affected by MS, and MS prevalence was detected in multi-aged native Chinese breeder chickens (3). Another study showed that 44,395 unvaccinated chickens were tested by ELISA from 21 provinces in China, and the overall seroprevalence rate was 41.19%, ranging from 5.10% to 100% in different provinces (15). Due to the interference of many factors, chickens immunized against the MS-H strain could not completely avoid infection with the field strains, so it is important to establish an accurate method to distinguish the MS-H vaccine strain from the field MS.

Various methods have been established to distinguish the MS-H strain and field strains, such as high-resolution melt-curve analysis (16), melt-curve and agarose gel-based mismatch amplification mutation assays (MAMA) (17), the qPCR method (9) based on SNPs in the *obg* gene, and nested PCR and high-resolution melt curve analysis (12). Furthermore, Kordafshari et al. developed indirect ELISA (18) based on nucleotide-shifting mutations in the *oppF* gene; target gene typing established by the sequence analysis of the PRR and RIII regions of the *vlhA* gene (19); and multilocus sequence typing (MLST) based on conserved housekeeping genes (20–22). Recent studies have shown that some of the MS-H isolates of the *obg* gene and *oppF* gene recovered the genotype or phenotype of the field 86079/7NS strain (23, 24). The conventional PCR sequencing method can discriminate between the MS-H stain and the field MS but has the disadvantage of being cumbersome and time-consuming, and the detection of some mixed samples of the MS-H strain and the field MS cannot be detected at the same time, which is also an important reason why it is not suitable for daily monitoring of commercial flocks.

In this study, we established a qPCR method with a pair of primers and two competitive TaqMan-MGB probes designed on the basis of SNP sites at 428 nt of the *hlyC* gene to discriminate between the MS-H stain and the field MS. This method has already been used for other pathogen detection (25). SNPs can undergo mutations, leading to false-positive and/or false-negative results, so additional evaluation of the results is needed whenever differential PCR assays for SNPs are widely used (9). For example, in the MS-H strain, the SNP site (A/G) at 367 nt in the *obg* gene appeared to revert to the parental strain 86079/7NS (24), and this phenomenon caused false-positive test results for field MS. Therefore, it is necessary to re-evaluate the results obtained by qPCR. To avoid errors and to further validate the accuracy of this method, sample sequencing can only confirm the results. Therefore, we performed PCR sequencing of a single MS-H strain or field MS sample DNA followed by sequencing. The sequencing results matched the qPCR results, indicating that our method is more accurate. Furthermore, compared with the above methods, the qPCR method has the characteristics of simple, fast, stronger sensitivity, or higher specificity.

The qPCR established in this study had 100% specificity and no cross-reactivity with MG, AIV, NDV, SE, IBV, *E. coli*, and Apg; the LOD of qPCR for the detection of the MS-H strain and the field MS was 1.07 × 10^1^ copies/µL and 1.95 × 10^1^ copies/µL, respectively. This highlights the good sensitivity of our qPCR method. This shows that the established qPCR could be used for the detection of clinical samples. Next, we tested 709 clinical samples from six poultry farms vaccinated with MS-H strains in Hebei province of China during 2022–2023. Only three farms were positive for field strains. Since Sanger sequencing could not identify mixed positive samples simultaneously, qPCR single positive samples were selected for further validation by *vlhA* gene sequencing. Successfully sequenced 335 single-MS-H positive samples and 146 single-field MS positive samples matched qPCR results, further demonstrating the potential of the qPCR method to distinguish MS field strains from MS-H strains. Interestingly, sequence analysis of the PRR region of the *vlhA* gene of the samples from farm A revealed three different nucleotide sequences, which were analyzed as MS-H strains, C-type field MS (XL12), and L-type field MS (XL13) (Fig. 4). It indicates that two different field MS infections were occurring on this immunized farm A against the MS-H strain. To the best of our knowledge, this is the first time that a C-type field MS has been detected by the *vlhA* gene analysis in China. This situation is similar to that reported by Catania (26), who reported that two different MS infections can coexist in the same flock. The difference is that we found that chickens immunized against the MS-H strain were simultaneously infected with two different field MS, and that three different MS strains coexisted in the flocks on farm A. The origin of the two field MS strains found in this study is unknown; they may have been introduced from different suppliers, or the chickens may have been infected by horizontal transmission, which will be investigated in future studies. This study only showed the presence of type C field strain infection and did not count the positive rate in farm A, which will be the next focus of the laboratory. The testing of clinical samples demonstrated that the qPCR developed can be used to monitor field strain infection in flocks immunized against the MS-H strain, which will guide us in MS prevention and control. There are no recognized and validated DIVA methods other than standard gene sequencing methods, making the results of this study incomparable, which is an important limitation of this study. The limited number of samples for clinical testing and sequencing is another important limitation of this study.

In conclusion, a TaqMan-qPCR method was established to discriminate between the MS-H strain and the field MS by the target *hlyC* gene, with the advantages of specificity, sensitivity, and repeatability. The study of clinical samples suggested the concurrent presence of L- and C-type field MS in immunized against MS-H flocks. This study provides a technical tool for clinical diagnosis and a reference base for further understanding the epidemiology of MS.

## MATERIALS AND METHODS

### Strains and samples

The field MS strains used in this study included the following: XL3 (GenBank accession no. CP133396), Hebei/2019/XJP (GenBank accession no. OP850570), Hebei/2019/HD-2 (GenBank accession no. OP850571), Hebei/2019/HD-4 (GenBank accession no. OP850573), Hebei/2020/HD-1 (GenBank accession no. OP850574), Hebei/2020/HD-2 (GenBank accession no. OP850575), Hebei/2022/HD-1 (GenBank accession no. OP850576), Hebei/2022/HD-2 (GenBank accession no. OP850577), Hebei/2022/HD-5 (GenBank accession no. OP850578), and Hebei/2022/HD-6 (GenBank accession no. OP850579). All isolated strains were identified and preserved at the Molecular Biology Laboratory of Hebei University of Engineering. Samples for clinical testing were collected from 6 large-scale chicken farms immunized against the MS-H strain vaccine in China; a total of 709 samples, the choanal cleft swabs, between and including the years 2022 and 2023.

### Analysis of the sequence and development of the differentiating MS qPCR

Based on the sequencing results of MS-H re-isolates compared with the parental strain 86079/7NS (23, 27, 28), at 428 nt of the hemolysin C (*hlyC*) gene (MS-H, CP021129, 481287 nt), the MS-H strain and its re-isolates were A and the parental strain 86079/7NS and other field MS were G, and it was hypothesized that this single nucleotide polymorphism (SNP) site might be a mutation site specific to the MS-H strain and its re-isolates (MS-H3, MS-H4, MS-H5, 101546, 101731, AS2, AB1, and TS4). To confirm the stability of this mutation on the MS-H strain, two TaqMan-MGB oligonucleotide probes and a pair of primers based on this SNP site (A/G) were designed in this study, and a real-time PCR method (qPCR) was set up to test the ability of the assay to discriminate between the MS-H-live vaccine strain and the field MS.

**Fig 5:**
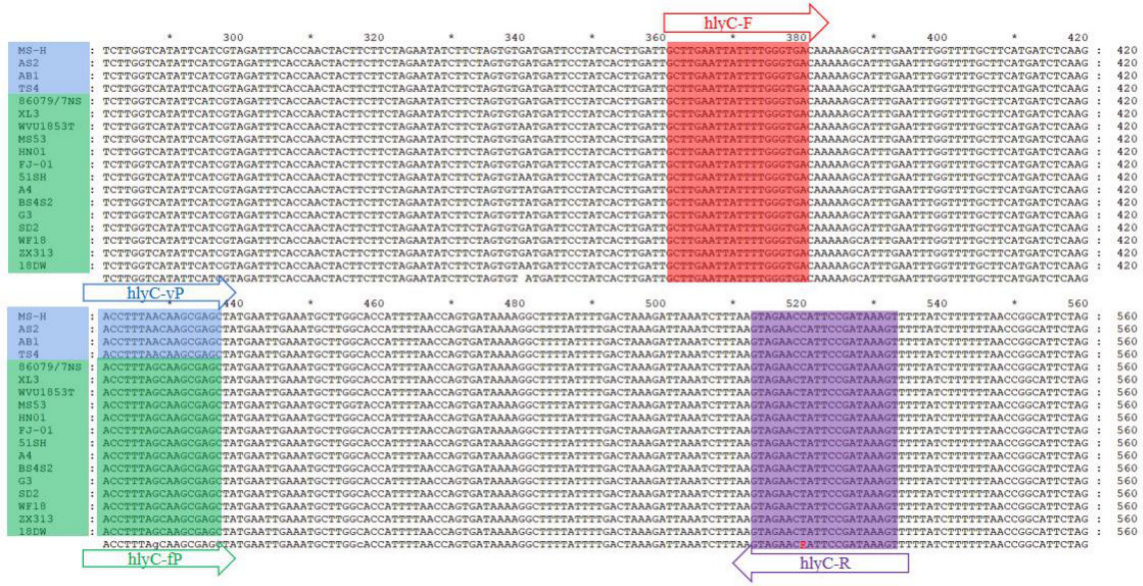
Sequence of primers and probes of qPCR. The forward primer sequence of the *hlyC* gene is denoted as hlyC-F (highlighted in red), and the reverse primer sequence is designated as hlyC-R (highlighted in purple), while the probe region for identifying MS-H and its re-isolates is labeled as hlyC-vP (highlighted in blue), while the probe area for detecting field strains is denoted by hlyC-fP (highlighted in green). The R of red is a concatenated base (C/T).

Genomic sequences of 18 MS strains published in GenBank were selected, the MS-H strain (CP021129) was used as a reference to select the *hlyC* gene 281–561 nt for sequence comparison using MEGA v7.0 and GeneDoc software (Fig. 5), and primers and probes were designed using the Primer Premier v5.0 software. The sequence information of the primers and probes is listed in Table 3, and all were produced by Kingsley Biotechnology Co. (Nanjing, China).

**TABLE 3:** Primer and probe sequence information^*a*^

Primer or probe	Sequence (5´→3´)	PCR product size (bp)
hlyC-F	GCTTGAATTATTTTGGGTGA	172
hlyC-R	CTTTATCGGAATRGTTCTACT	
hlyC-vP	FAM-ACCTTTA**A**CAAGCGAGC-MGB	
hlyC-fP	VIC-ACCTTTA**G**CAAGCGAGC-MGB	

^*a*^ SNPs between MS-H vaccine strains and field strains are indicated in bold.

The final reaction volume for the qPCR was 20 µL according to the instructions for the 2× AceQ qPCR Probe Master Mix (Vazyme, Nanjing, China). The multiplex reaction system was then optimized using different volumes (0.3 µL, 0.4 µL, 0.5 µL, 0.6 µL, and 0.7 µL) of primers (10 µM) and probes (10 µM), and the optimal volumes of templates were determined. The plasmid standards containing 1 × 10^3^ copies/μL were chosen as templates. Optimization of the annealing temperature based on the estimated Tm values was performed at the following temperatures: 56°C, 58°C, 60°C, 62°C, and 64°C. The optimal reaction system was as follows: hlyC-qPCR reaction system: 2× AceQ qPCR Probe Master Mix (Vazyme, Nanjing, China) 10 µL, 50× ROX Reference Dye 2 (Vazyme, Nanjing, China) 0.4 µL, hlyC-F (10 µM) 0.6 µL, hlyC-R (10 µM) 0.6 µL, hlyC-vP (10 µM) 0.5 µL, hlyC-fP (10 µM) 0.5 µL, template DNA solution 2 µL, and sterile deionized water 5.4 µL. Reactions were carried through in the ABI 7500 real-time PCR instrument (Thermo Fisher Scientific, America) with the following programs: 95°C for 5 min, followed by 40 cycles of 95°C for 10 s and 60°C for 34 s.

### Construction of recombinant plasmids

For the strains used in this study, the XL3 field strain isolated in our laboratory and the live vaccine MS-H strain (Vaxsafe®, Australia) were used as positive controls. The DNA solutions of MS field strain XL3 and MS-H vaccine strains were extracted using the Bacterial Genomic DNA Rapid Extraction Kit (Sangon Biotech, Shanghai, China).

PCR amplification was performed using primers hlyC-F/hlyC-R for DNA solutions of XL3 and MS-H strains. The PCR assay was performed in a 50 µL reaction volume consisting of a 2 µL DNA template; 1 µL primers (10 µM); and 25 µL 2× Taq PCR StarMix (Vazyme, Nanjing, China). Reactions were carried through in a PCR instrument (Thermo Fisher Scientific, America) with the following programs: 95°C for 5 min; followed by 32 cycles of 95°C for 30 s, 55°C for 30 s, 72°C for 20 s, and 72°C for 10 min. PCR products were purified using the FastPure Gel DNA Extraction Mini Kit (Vazyme, Nanjing, China) and cloned into the pMD19-T vector (Takara, Beijing, China) to construct recombinant plasmids (pMD19-T/MS-H, pMD19-T/XL3), which were obtained from *E. coli* DH5α cells (Vazyme, Nanjing, China) by EndoFree Plasmid Mini Kit (Cwbio, Jiangsu, China). Recombinant plasmids were successfully validated by sequencing (Kingsley Biotechnology, Nanjing, China). The concentration of these plasmids was expressed as the number of copies per µL using the following formula: y (copies/µL) = (6.02 × 10^23^) × (× (ng/µL) × 10^−9^DNA)/(DNA length × 660) (29) and stored at −20°C.

### qPCR analytical sensitivity, amplification efficiency, and standard curves

The recombinant plasmids were used with enzyme-free sterile water to dilute 10-fold in the range of 10^10^–10° copies/μL to verify the sensitivity of the qPCR. The positive plasmids underwent sequencing verification and were utilized as standard positive controls to develop quantitative analysis standard curves.

The final concentration of plasmids was made up to 10^9^ copies/µL and serially diluted 10-fold with nuclease-free water. Standard curves were generated using 10× serial dilutions of the standard plasmids, and correlation coefficient (*R*^2^) values and amplification efficiency (*E*%) were calculated.

### Specificity assessment

The primers were searched in the nucleotide collection (nt) database of primer blast to analyze their specificity. A specificity test was performed by the qPCR method using DNA or cDNA of field MS strains, MS-H strains, MG, *Salmonella enterica* (SE), *avian influenza virus* (AIV), *Newcastle disease virus* (NDV)*, infectious bronchitis virus* (IBV), *E. coli*, and *Avibacterium paragallinarum* (Apg). The 10 MS field strains (XL3, Hebei/2019/XJP, Hebei/2019/HD-2, Hebei/2019/HD-4, Hebei/2020/HD-1, Hebei/2020/HD-2, Hebei/2022/HD-1, Hebei/2022/HD-2, Hebei/2022/HD-5, and Hebei/2022/HD-6) and MS-H strains were used as a positive control for the field strain and the vaccine strain, and ddH_2_O was used as a negative control. The DNA or cDNA of these pathogens was kept and provided by the Molecular Biology Laboratory of Hebei University of Engineering.

### Reproducibility assessment

Repeatability testing was performed by selecting dilutions of plasmid standards with concentrations of 10^5^, 10^4^, and 10^3^ for qPCR amplification. These were extracted and tested with a single extraction performed. Intra-assays were tested using the same batch of reagents prepared in triplicate by the same operator, and inter-assays were tested by three operators who prepared the different batches of reagents separately. The Cq values for samples of varying concentrations were tested three times to calculate their reproducibility and the coefficient of variation (%CV).

### Clinical sample testing

The 709 samples were subjected to qPCR. All samples were subjected to PCR of the *vlhA* gene primers (link: TACTATTAGCAGCTAGTGC, MSConsR: AGTAACCGATCCGCTTAAT) (30), and the positive products were verified by Sanger sequencing (Saiheng Biotechnology, Shanghai, China). The nucleotide insertion/deletion of the proline-rich repeat (PRR) region and the nucleotide polymorphisms of the RIII region in *vlhA* gene fragments were the basis for the *vlhA* gene sequence analysis, which was helpful for MS strain typing and subtyping (19). This method can distinguish MS-H strains from field strains based on sequence differences (30).
